# Clinicopathological and molecular characterisation of ‘multiple‐classifier’ endometrial carcinomas

**DOI:** 10.1002/path.5373

**Published:** 2020-01-12

**Authors:** Alicia León‐Castillo, Ester Gilvazquez, Remi Nout, Vincent THBM Smit, Jessica N McAlpine, Melissa McConechy, Stefan Kommoss, Sara Y Brucker, Joseph W Carlson, Elisabeth Epstein, Tilman T Rau, Robert A Soslow, Raji Ganesan, Xavier Matias‐Guiu, Esther Oliva, Beth T Harrison, David N Church, C Blake Gilks, Tjalling Bosse

**Affiliations:** ^1^ Department of Pathology Leiden University Medical Center Leiden The Netherlands; ^2^ Wellcome Centre for Human Genetics University of Oxford Oxford UK; ^3^ National Institute for Health Research (NIHR) Oxford Biomedical Research Centre Oxford University Hospitals NHS Foundation Trust, John Radcliffe Hospital Oxford UK; ^4^ Department of Medical and Radiation Oncology Leiden University Medical Center Leiden The Netherlands; ^5^ Department of Gynecology, Division of Gynecologic Oncology University of British Columbia and BC Cancer Agency Vancouver Canada; ^6^ Contextual Genomics Inc Vancouver Canada; ^7^ Department of Women's Health Tübingen University Hospital Tübingen Germany; ^8^ Department of Oncology–Pathology, Karolinska Institutet, and Department of Pathology and Cytology Karolinska University Hospital Stockholm Sweden; ^9^ Department of Clinical Science and Education Karolinska Institutet, and Department of Obstetrics and Gynaecology Södersjukhuset Stockholm Sweden; ^10^ Institute of Pathology University of Bern Bern Switzerland; ^11^ Department of Pathology Memorial Sloan Kettering Cancer Center New York NY USA; ^12^ Department of Pathology Birmingham Women's NHS Foundation Trust Birmingham UK; ^13^ Department of Pathology, Hospital U Arnau de Vilanova and Hospital U de Bellvitge Universities of Lleida and Barcelona, IDIBELL, IRBLLEIDA, CIBERONC Lleida Spain; ^14^ Department of Pathology, Massachusetts General Hospital Harvard University Boston MA USA; ^15^ Department of Pathology Brigham and Women's Hospital Boston MA USA; ^16^ Department of Pathology and Laboratory Medicine University of British Columbia and Vancouver General Hospital Vancouver Canada

**Keywords:** POLE, molecular classification, endometrial cancer

## Abstract

Endometrial carcinoma (EC) molecular classification based on four molecular subclasses identified in The Cancer Genome Atlas (TCGA) has gained relevance in recent years due to its prognostic utility and potential to predict benefit from adjuvant treatment. While most ECs can be classified based on a single classifier (*POLE* exonuclease domain mutations – *POLE*mut, MMR deficiency – MMRd, p53 abnormal – p53abn), a small but clinically relevant group of tumours harbour more than one molecular classifying feature and are referred to as ‘multiple‐classifier’ ECs. We aimed to describe the clinicopathological and molecular features of multiple‐classifier ECs with abnormal p53 (p53abn). Within a cohort of 3518 molecularly profiled ECs, 107 (3%) tumours displayed p53abn in addition to another classifier(s), including 64 with MMRd (MMRd–p53abn), 31 with *POLE*mut (*POLE*mut–p53abn), and 12 with all three aberrations (MMRd–*POLE*mut–p53abn). MMRd–p53abn ECs and *POLE*mut–p53abn ECs were mostly grade 3 endometrioid ECs, early stage, and frequently showed morphological features characteristic of MMRd or *POLE*mut ECs. 18/28 (60%) MMRd–p53abn ECs and 7/15 (46.7%) *POLE*mut–p53abn ECs showed subclonal p53 overexpression, suggesting that *TP53* mutation was a secondary event acquired during tumour progression. Hierarchical clustering of TCGA ECs by single nucleotide variant (SNV) type and somatic copy number alterations (SCNAs) revealed that MMRd–p53abn tumours mostly clustered with single‐classifier MMRd tumours (20/23) rather than single‐classifier p53abn tumours (3/23), while *POLE*mut–p53abn tumours mostly clustered with single‐classifier *POLE*mut tumours (12/13) and seldom with single‐classifier p53abn tumours (1/13) (both *p* ≤ 0.001, chi‐squared test). Finally, the clinical outcome of patients with MMRd–p53abn and *POLE*mut–p53abn ECs [stage I 5‐year recurrence‐free survival (RFS) of 92.2% and 94.1%, respectively] was significantly different from single‐classifier p53abn EC (stage I RFS 70.8%, *p* = 0.024 and *p* = 0.050, respectively). Our results support the classification of MMRd–p53abn EC as MMRd and *POLE*mut–p53abn EC as *POLE*mut. © 2019 The Authors. *The Journal of Pathology* published by John Wiley & Sons Ltd on behalf of Pathological Society of Great Britain and Ireland.

## Introduction

Of the many advances in the field of endometrial cancer (EC) during the last decade, perhaps the one with most impact is the molecular classification proposed by The Cancer Genome Atlas (TCGA) 1, which has gained prominence in recent years 2. This classifies ECs into four molecular subtypes – *POLE*/ultramutated (*POLE*), microsatellite instability‐high/hypermutated (MSI), somatic copy‐number alteration high/serous‐like (SCNA‐high), and somatic copy‐number alteration low (SCNA‐low) – with significantly different prognoses 3, and thus of potential clinical relevance. For example, the favourable outcome of *POLE* (exonuclease domain) mutant EC, independent of adjuvant treatment 4, has led to proposals to de‐escalate post‐operative therapy in this subgroup 4. In contrast, the consistently poor clinical outcome of patients with SCNA‐high (serous‐like) ECs suggests that intensification of adjuvant treatment may be worthwhile. The reported response of MSI tumours to immune checkpoint inhibitors 5, 6, as well as the possibility of a better response to adjuvant radiotherapy 7, also opens new treatment opportunities for this group of patients. A randomised controlled clinical trial (PORTEC4a 8) is currently testing the added value of integrating this molecular approach into risk assessment, to individualise adjuvant treatment and reduce over‐ and under‐treatment in patients 9.

One important contributor to the impact and rapidly increasing adoption of the TCGA classifier is the relative ease in which subgroups analogous to those originally described can be identified by techniques in routine clinical practice 9, 10, 11, 12, 13, 14. ECs analogous to the SCNA‐high subclass can be identified by p53 immunohistochemistry (p53abn EC) 15, the MSI subclass by immunohistochemistry for MMR proteins (MMRd EC), and the *POLE* subclass by targeted sequencing of the *POLE* exonuclease domain (*POLE*mut EC) (the latter being the most difficult to implement in routine practice). Tumours lacking these three prior features are classified as p53 wild‐type (p53wt EC) 11 or no specific molecular profile subtype (NSMP EC) 10, analogous to the SCNA‐low subclass.

Using this surrogate marker approach, most ECs can be classified into a single molecular class (henceforward referred to as ‘single‐classifier’ ECs). However, 3–6% of tumours have more than one molecular classifying feature (hereafter referred to as ‘multiple‐classifier’ ECs) 9, 10, 11, 12, and include those with combined *POLE* exonuclease domain mutation (EDM) and abnormal p53 (*POLE*mut–p53abn), combined DNA mismatch repair deficiency (MMRd) and abnormal p53 (MMRd–p53abn), combined MMRd and *POLE* EDM (MMRd–*POLE*mut), and all three defects (MMRd–*POLE*mut–p53abn) 9, 10, 11, 12. There is currently no consensus on how these tumours should be classified or treated; some studies have excluded them from further analysis 10, while others have allocated them to one of the four subtypes 11 without detailing their clinicopathological or molecular features. Consequently, the biology and prognostic significance of multiple‐classifier ECs are unclear, and how they should be managed is unanswered. This creates an important problem for tumours that carry opposite features, as one may favour treatment de‐escalation (*POLE*mut) and the other intensified treatment (p53abn).

In this study, we aimed to perform a comprehensive analysis of the clinical, morphological, and molecular characteristics of EC with abnormal p53 immunostaining and/or *TP53* mutation in combination with *POLE*mut and/or MMRd in order to inform clinical management of these multiple‐classifier ECs.

## Materials and methods

### Patient and tissue selection

Patient identity was protected by study‐specific patient numbers. Informed consent and ethical approval were obtained according to the local protocol in each participating centre.

We obtained patient data and tumour tissue from ECs with more than one classifying feature (a pathogenic *POLE* exonuclease domain variant, MMR protein loss of expression or p53 abnormal expression) from previously published datasets 1, 10, 12, 13, 16, 17. A total of 2988 ECs had been molecularly classified in previous studies 10, 12, 13, 16, 17, in which MMR and p53 status were determined by immunohistochemistry (IHC) and *POLE* variants by Sanger sequencing or NGS of the complete exonuclease domain (exons 9–14) or targeted sequencing of exons 9, 13, and 14. For this current study, all *POLE* variants were reviewed and tumours were excluded when the *POLE* variant was not considered pathogenic following the recommended approach presented by León‐Castillo *et al* 18. Additionally, following the criteria mentioned previously, four tumours reported as having all three molecular features (*POLE*mut–MMRd–p53abn) but with a non‐pathogenic *POLE* EDM based on review were classified as MMRd–p53abn ECs. ECs with MMR loss and a pathogenic *POLE* variant (MMRd–*POLE*mut ECs) were excluded from this study, as they are described by León‐Castillo *et al* 18. Clinical follow‐up and, when available, slides were centrally collected for further analyses. Follow‐up data were provided by each centre in an anonymised dataset and datasets were combined into a final password‐protected database.

Our study cohort was further extended by 530 TCGA ECs in which a combination of a pathogenic *POLE* EDM, a *TP53* variant (excluding *TP53* variants classified as benign by SIFT or neutral by VEP), and/or MSI‐H (based on Bethesda protocol classification 19 was obtained from the Genome Data Analysis Center (GDAC) (available at http://www.broadinstitute.org/cancer/cga). *TP53* mutations were assessed using the public databases COSMIC 20, ClinVar 21, and IARC TP53 mutations database 22, as well as the *in silico* tools SIFT 23 and PolyPhen 24; only mutations classified as (likely) pathogenic or variants of unknown significance (VUS) were included in the study. This resulted in a final study cohort of 3518 molecularly profiled ECs.

### Scoring MMR and p53 IHC

As multiple‐classifier ECs are identified by IHC, we re‐evaluated or restained tumours with available unstained slides to exclude potential misinterpretation in the original study. Unstained sections were stained for either two (PMS2 and MSH6) or four mismatch repair proteins (MLH1, PMS2, MSH2, and MSH6) dependent on slide availability [MLH1 (ES05, 1:100; Agilent DAKO, Amstelveen, North Holland, The Netherlands), PMS2 (EP51, 1:50; Agilent DAKO), MSH2 (FE11, 1:200; Agilent DAKO), and MSH6 (EPR 3945, 1:800; GeneTex, Irvine, CA, USA)] and p53 (DO‐7, 1:200; Agilent DAKO).

MMRd was defined as loss of MMR nuclear staining, for at least one MMR protein, with positive internal control. Subclonal loss of MMR expression 25 was defined as abrupt and complete regional loss of expression of an MMR protein with intervening stromal positivity serving as an internal control in the regions of absent tumour cell staining. Cases with heterogeneous or non‐abrupt patchy staining thought to be a result of sub‐optimal pre‐analytic handling or those with absent internal control staining were not considered MMRd. An abnormal/mutant p53 IHC stain was defined as strong nuclear expression in over 75% of the tumour cells (overexpression), complete loss of nuclear stain in the presence of internal control staining (complete absence), or cytoplasmic staining (cytoplasmic), following scoring described by Singh *et al* 15. Subclonal abnormal p53 IHC staining was defined as abrupt and complete regional abnormal p53 expression, in which the subclonal region was at least 10% of the total tumour volume. If the p53 IHC staining pattern could not be classified into one of the previously mentioned patterns (inconclusive p53 stain), *TP53* mutational status was used for molecular profiling through Sanger sequencing of exons 5–8.

### Histopathological review

Tumours with at least one haematoxylin–eosin (H&E) slide available were selected for histological review, blinded for molecular classification, by one pathologist (AL, TB, BG or RS). This review consisted of scoring for each case predefined histological features: hobnailing, slit‐like spaces, papillary growth, squamous metaplasia, tumour intraepithelial lymphocytes (TILs), peritumoural lymphocytes, solid growth greater than 50%, and the presence of tumour giant cells. For the purpose of this study, we did not reassess tumour histotype. Type of myometrial invasion and presence of lymphovascular space invasion (LVSI) were also annotated.

### Evaluation of somatic nucleotide and copy number variation in TCGA ECs

Somatic mutations were classified into the 96 categories described by Alexandrov *et al* 26. Distance metrics for the difference between pairs of samples were generated using the mutational changes (1 – cosine similarity/2) or the proportion of genome that had a copy number change (both of these range between 0 and 1). These distances were combined into a single metric using Euclidean distance. Individual and combined metrics were used to cluster the samples and plot a heatmap using the function heatmap.2 from the package ‘gplots’ to visualise similarity between samples.

### Statistical analysis

We assessed 5‐year recurrence‐free survival (RFS) and 5‐year overall survival (OS) comparing Kaplan–Meier curves with the log‐rank test. Follow‐up time was calculated with the reverse Kaplan–Meier method. We used Mann–Whitney tests to compare non‐parametric continuous variables; categorical variables were assessed with Fisher's exact test or the chi‐squared test. Two‐sided *P* value less than 0.05 was considered significant.

## Results

### Identification and clinicopathological characteristics of multiple‐classifier ECs

**Figure 1:**
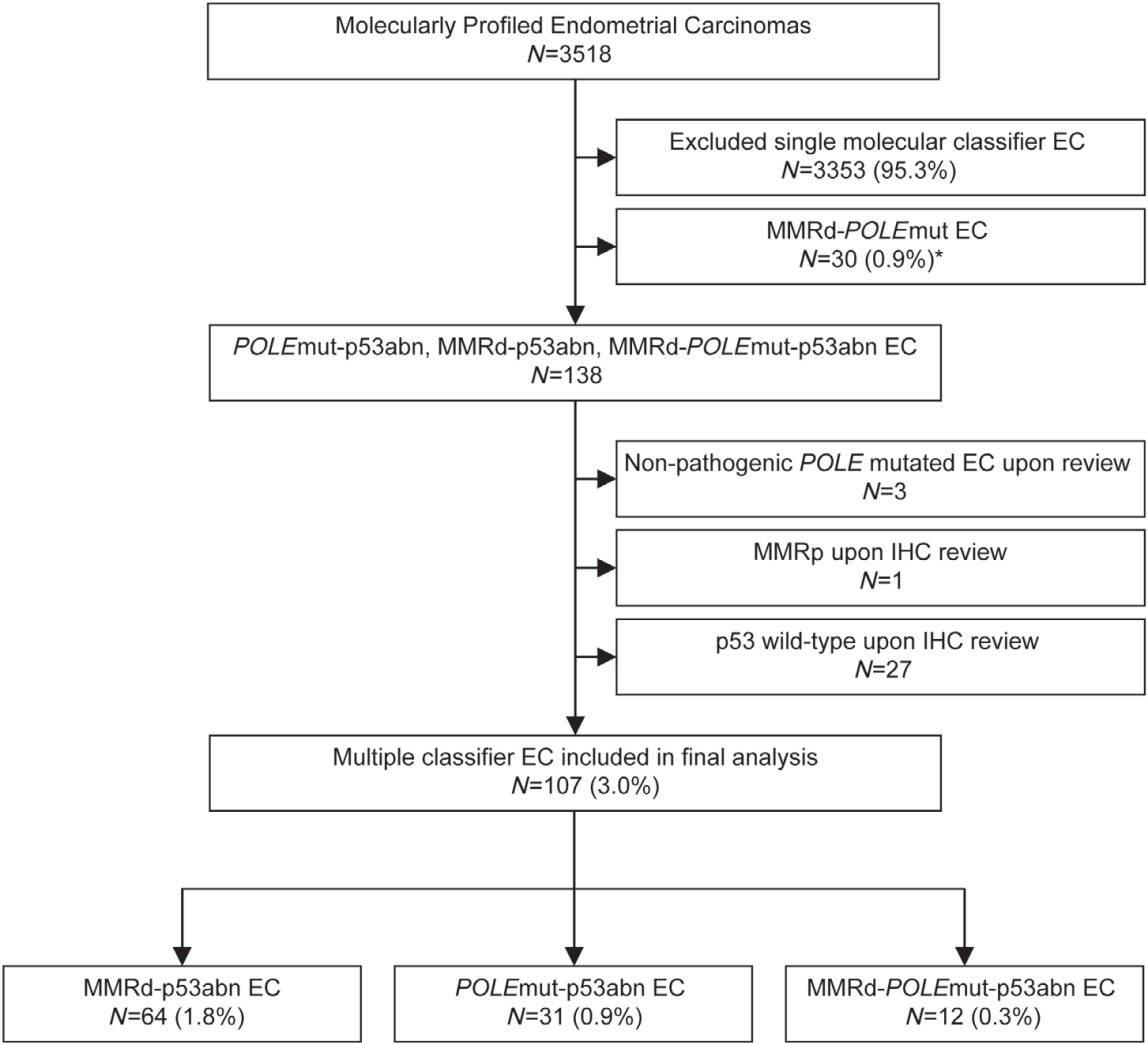
Flow chart of sample analysis. *POLE*mut–MMRd ECs are reported separately in León‐Castillo *et al* 18.

Our initial cohort comprised 3518 tumours, 3353 being classified to a single molecular subtype. Of the remaining 167 tumours, 30 were classified as MMRd–*POLE*mut ECs and will be reported separately (León‐Castillo *et al* 18), leaving 138 tumours (3.9%) that were assigned a provisional status of multiple‐classifier with abnormal p53 (mutant p53 expression by IHC or a mutation in *TP53*). Stringent quality control including central pathological review resulted in the exclusion of 35 of these ECs for the following reasons: (1) reassignment of p53 immunostaining from mutant to wild‐type pattern (*n* = 27, 21 being initially evaluated using tissue microarrays); (2) non‐pathogenic *POLE* variant (*n* = 3); and (3) lack of evidence of MMRd on IHC review (*n* = 1) (Figure 1). The remaining 107 tumours (3% of total) met the inclusion criteria and were used for subsequent analyses.

The clinicopathological characteristics of the patients with these 104 ECs are shown in Table 1 27, 28. The mean age was 62 (range 35–87) years. The FIGO 2009 stage was as follows: 80 (76.9%) stage I and 24 (23.1%) stage II–IV. The most common combination of classifiers was MMRd–p53abn (64; 1.8% of 3518), followed by *POLE*mut–p53abn (31; 0.9% of 3518) and MMRd–*POLE*mut–p53abn (12; 0.3% of 3518).

**Table 1:** Clinicopathological characteristics of multiple‐classifier EC with abnormal p53

	Total *n* = 107 (%)	MMRd–p53abn EC *n* = 64 (%)	*POLE*mut–p53abn EC *n* = 31 (%)	MMRd–*POLE*mut–p53abn EC
				*n* = 12 (%)
Age, years				
Mean [range]	61.6 [35–87]	61.7 [35–87]	62.1 [50–83]	59.9 [47–74]
< 60	51 (47.7)	28 (43.8)	16 (51.6)	7 (58.3)
60–70	33 (30.8)	22 (34.4)	8 (25.8)	3 (25.0)
> 70	22 (20.6)	14 (21.9)	6 (19.4)	2 (16.7)
Missing	1 (0.9)	0 (0)	1 (3.2)	0 (0)
Stage				
IA	41 (38.3)	25 (39.1)	9 (29)	7 (58.3)
IB	41 (38.3)	22 (34.4)	15 (48.4)	4 (33.3)
II	3 (2.8)	2 (3.1)	1 (3.2)	0 (0)
III	16 (15)	11 (17.2)	4 (12.9)	1 (8.3)
IV	6 (5.6)	4 (6.3)	2 (6.5)	0 (0)
Histology				
Endometrioid	77 (72)	46 (71.9)	22 (71)	9 (75.0)
Serous	9 (8.4)	6 (9.4)	2 (6.5)	1 (8.3)
Mixed	16 (15)	8 (12.5)	6 (19.4)	2 (16.7)
Clear cell	3 (2.8)	2 (3.1)	1 (3.2)	0 (0)
Undifferentiated	2 (1.9)	2 (3.1)	0 (0)	0 (0)
Grade				
1–2	25 (23.4)	16 (25)	7 (22.6)	2 (16.7)
3	82 (76.6)	48 (75)	24 (77.4)	10 (83.3)
Myometrium invasion				
Intramucosal	4 (3.7)	2 (3.1)	0 (0)	2 (16.7)
< 50%	45 (42.1)	28 (43.8)	11 (35.5)	6 (50.0)
> 50%	53 (49.5)	31 (48.4)	19 (61.3)	4 (33.3)
Missing	5 (4.7)	3 (4.7)	1 (3.2)	0 (0)
LVSI				
Absent	44 (41.1)	22 (34.4)	15 (48.4)	7 (58.3)
Present	32 (29.9)	23 (35.9)	5 (16.1)	4 (33.3)
Missing	31 (29.0)	19 (29.7)	11 (35.5)	1 (8.3)
Treatment				
Radiotherapy	18 (16.8)	12 (18.8)	5 (16.1)	1 (8.3)
Chemotherapy	9 (8.4)	4 (6.3)	4 (12.9)	1 (8.3)
Radiochemotherapy	10 (9.3)	7 (10.9)	3 (9.7)	0 (0)
None	15 (14.0)	9 (14.1)	4 (12.9)	2 (16.7)
Missing	55 (51.4)	32 (50)	15 (48.4)	8 (66.7)
Risk classification (ESMO clinical practice guidelines, 2013 27)				
Low risk	9 (8.4)	6 (9.4)	3 (9.7)	0 (0)
Intermediate risk	31 (29.0)	16 (25)	8 (25.8)	7 (58.3)
High risk	51 (47.7)	30 (46.9)	17 (54.8)	4 (33.3)
Advanced stage I	13 (12.1)	10 (15.6)	2 (6.5)	1 (8.3)
Metastatic	3 (2.8)	2 (3.1)	1 (3.2)	0 (0)
Risk classification (ESMO–ESTRO–ESGO clinical practice guidelines, 2016 28)				
Low risk	4 (3.7)	2 (3.1)	2 (6.5)	1 (8.3)
Intermediate	11 (10.3)	7 (10.9)	3 (9.7)	0 (0)
High–intermediate	22 (20.6)	11 (17.2)	5 (16.1)	6 (50)
High	59 (55.1)	37 (57.8)	17 (54.8)	5 (41.7)
Advanced or metastatic	6 (5.6)	4 (6.3)	2 (6.5)	0 (0)
Not assessable	5 (4.7)	3 (4.7)	2 (6.5)	0 (0)

### DNA mismatch repair‐deficient, p53 abnormal (MMRd–p53abn) ECs

From the total study population, 64 tumours had both MMRd/MSI and abnormal p53 (MMRd–p53abn) (Table 1). Of these, 40 (62.5%) were classified based on MMR protein expression, with an unusually high number of ECs (*n* = 25, 62.5%) presenting with loss of MSH6 +/− MSH2, or single PMS2 loss 25. The mean patient age in this subgroup was 62 years. Most tumours were endometrioid (46/64, 71.9%), with 65.2% being grade 3. 76.7% (49/64) of these patients had early‐stage ECs (stage I–II). Central histological review was possible in 54 ECs (median of two slides per case, range 1–17) (supplementary material, Table S1) and typically revealed a dense lymphoid infiltrate (peritumoural lymphocytes in 59.3% and tumour‐infiltrating lymphocytes in 42.6% of tumours). LVSI was observed in 33.3% of tumours. Solid growth (≥ 50% of the tumour) was present in 24 (44.4%) ECs, and squamous metaplasia was identified in 13 (24.1%). Hobnailing, slit‐like spaces or papillary growth was present in eight (14.8%), 12 (22.2%), and 14 (25.9%) tumours, respectively.

**Figure 2:**
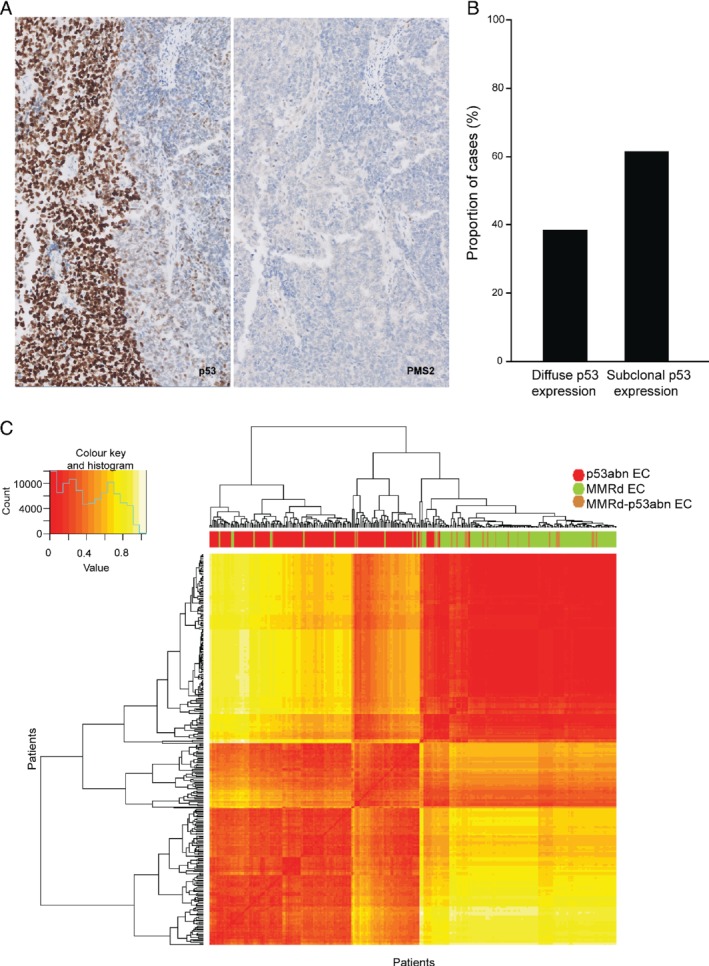
MMRd–p53abn EC IHC and molecular features. Subclonal p53 staining and loss of PMS2 are shown in A (×5 original magnification). Percentage of cases with subclonal and diffuse abnormal p53 staining patterns is depicted in B. (C) Heatmap showing hierarchical clustering of MMRd–p53abn, single‐classifier MMRd, and single‐classifier p53abn ECs in TCGA, based on mutational changes and copy number changes. Individual similarity metrics for copy number and mutational changes were combined using Euclidean distance. Patients were classified in groups based on MMRd–p53abn (brown), single‐classifier MMRd (green), and single‐classifier p53abn (red). Samples were ordered based on hierarchical clustering.

p53 IHC was available for central review in 28 of the 64 MMRd–p53abn tumours. Nine (30%) showed p53 overexpression, while one tumour had an inconclusive staining pattern in combination with a confirmed pathogenic *TP53* mutation. Interestingly, the remaining 18 tumours (60%) showed abrupt strong p53 nuclear overexpression (> 75% of all tumour nuclei) in a well‐defined area (at least 10% of tumour volume), a pattern defined as subclonal p53abn staining (Figure 2).

To further characterise the group of MMRd–p53abn ECs, we used sequencing data from the TCGA study, comparing analogous tumours with combined microsatellite instability and *TP53* mutation (MMRd–p53abn EC, *n* = 23) with those with one of these defects in isolation (henceforth referred to as single‐classifier MMRd EC if microsatellite‐unstable and single‐classifier p53abn EC if *TP53*‐mutant). Fifteen (65%) MMRd–p53abn ECs had a (likely) pathogenic *TP53* mutation and eight (35%) had a *TP53* VUS. Preliminary analysis revealed a significantly higher frequency of multiple *TP53* mutations in MMRd–p53abn tumours when compared with single‐classifier p53abn (i.e. lacking *POLE* mutation or MSI‐H) ECs (36.4% versus 2.7%, *p* < 0.001, Fisher's exact test). To further define the molecular characteristics of MMRd–p53abn ECs, we performed hierarchical clustering of TCGA ECs according to the proportion of SNVs of each trinucleotide context and SCNA burden (see the Materials and methods section), revealing that MMRd–p53abn ECs clustered mostly with single‐classifier MMRd tumours (which are typically SCNA‐low) rather than with single‐classifier p53abn ECs (20/23 versus 3/23, respectively; *p* ≤ 0.001) (Figure 2). Similar results were obtained when analysing individually SNVs and SCNA (supplementary material, Figure S1).

### *POLE*‐mutant, p53‐abnormal (*POLE*mut–p53abn) ECs

A total of 31 ECs (0.9%) had both a *POLE* mutation and abnormal p53 (*POLE*mut–p53abn) (Table 1). In all of these *POLE*mut–p53abn ECs, the *POLE* variant was a known pathogenic mutation including p.Pro286Arg (16), p.Val411Leu (10), Ala456Pro (3), p.Ser459Phe (1), and p.Pro436Arg (1), as described by León‐Castillo *et al* 18. Patients with *POLE*mut–p53abn ECs had a mean age of 62 years. Histologically, these tumours were most frequently grade 3 endometrioid EC (*n* = 15, 48.4%). Twenty‐four patients had (77.4%) stage I tumours, 1 (3.2%) had a stage II, 4 (12.9%) had stage III, and 2 had (6,5%) stage IV tumours. Thirty cases were available for morphological review (median two slides available per case, range 1–15) (supplementary material, Table S1). This revealed a prominent lymphoid infiltrate in the majority of ECs (90.9% with peritumoural lymphocytes and 59.1% with TILs). Tumour giant cells, previously reported in 33–40% of *POLE*‐mutant single‐classifier ECs 29, 30, were observed in 12 (40%) tumours and substantial LVSI was present in 10%. Solid growth ≥ 50% was identified in 16 cancers (53.3%) and squamous metaplasia in 4 (13.3%). Hobnailing was observed in four (13.3%), slit‐like spaces in six (20%), and papillary growth in four (13.3%) tumours.

**Figure 3:**
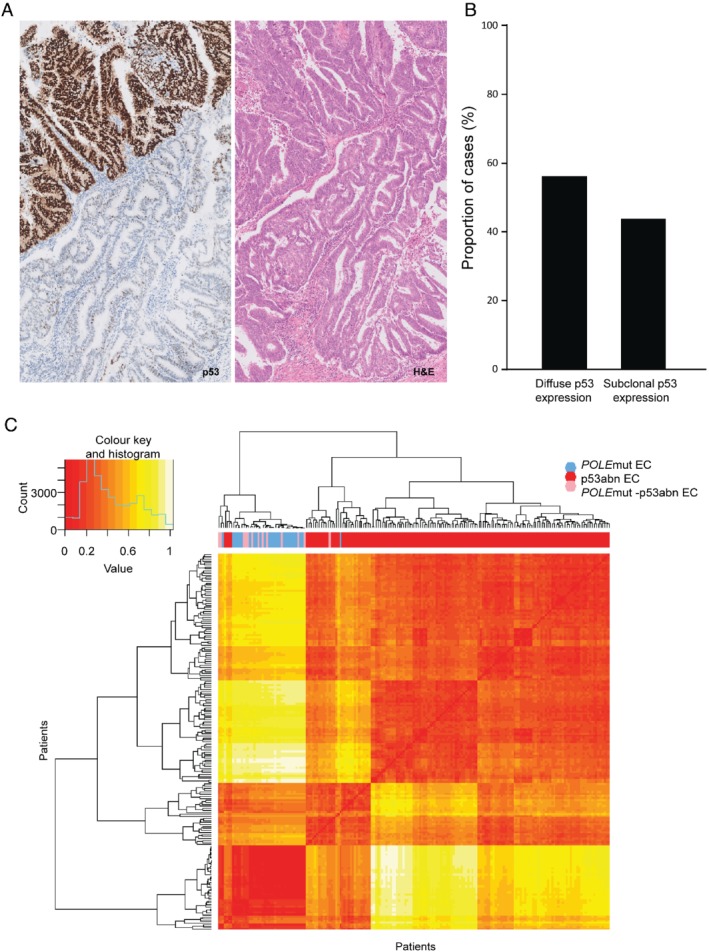
*POLE*mut–p53abn EC IHC and molecular features. Subclonal p53 staining and H&E stain of a *POLE*mut–p53abn EC are shown in A (×5 original magnification). Percentage of cases with subclonal and diffuse abnormal p53 staining patterns is depicted in B. (C) Heatmap showing hierarchical clustering of *POLE*mut–p53abn, single‐classifier *POLE*mut, and single‐classifier p53abn ECs in TCGA, based on mutational changes and copy number changes. Individual similarity metrics for copy number and mutational changes were combined using Euclidean distance. Patients were classified in groups based on *POLE*mut–p53abn (pink), single‐classifier *POLE*mut (blue), and single‐classifier p53abn (red). Samples were ordered based on hierarchical clustering.

Central review of p53 immunostaining was possible in 15 tumours. Six (37.5%) had diffuse overexpression of p53; one showed complete loss of p53 expression; and one had inconclusive p53 staining with a confirmed *TP53* mutation. Intriguingly, similar to our results in MMRd–p53 multiple‐classifier ECs, subclonal abnormal p53 staining (subclonal overexpression) was frequent (*n* = 7, 46.7%) (Figure 3).

We used the TCGA EC cohort to compare the 13 ECs with combined *POLE* mutation and *TP53* mutation (*POLE*mut–p53abn) with tumours with either defect alone (single‐classifier *POLE*mut and single‐classifier p53abn EC). Eight (62%) *POLE*mut–p53abn ECs had at least one *TP53* (likely) pathogenic mutation, the remaining five (38%) having at least one VUS. *POLE*mut–p53abn ECs more frequently had multiple *TP53* mutations when compared with single‐classifier p53abn tumours (75% versus 2.7%, *p* < 0.001, Fisher's exact test*)*. Additionally, known *TP53* mutational hot‐spot codons Arg 175, Gly 245, Arg 248, Arg 249, Arg 273, and Arg 282 31 were seldom mutated in *POLE*mut–p53abn ECs, in contrast to single‐classifier *TP53*mut ECs where these alterations were common, although this difference was not statistically significant [1/12 (8%) versus 57/184 (31.5%); *p* = 0.11].

Further analysis of *TP53* mutations by trinucleotide context demonstrated that TCT>TAT alterations (characteristic of *POLE*mut carcinomas 26, 32) were uncommon in *POLE*mut–p53abn ECs (4.2%) and absent in single‐classifier p53abn ECs (0/116) (*p =* 0.31). However, TCG>TTG substitutions (also substantially enriched in *POLE*mut carcinomas 32) accounted for 6/24 (25%) of the *TP53* variants in *POLE*mut–p53abn in contrast to single‐classifier p53abn ECs in which these alterations did not occur (*p* ≤ 0.001*)*.

Hierarchical clustering of ECs by SNV and SCNA proportions revealed that 12 of 13 *POLE*mut–p53abn ECs clustered with single‐classifier *POLE‐*mutant ECs, while a single case clustered with the single‐classifier p53abn tumours (*p* ≤ 0.001, chi‐squared test) (Figure 3). Separate analysis of SNV and SCNAs rendered similar results (supplementary material, Figure S2).

### *POLE*‐mutant, DNA mismatch repair‐deficient p53‐abnormal (MMRd–*POLE*mut–p53abn) ECs

From our pooled cohort, 12 ECs (0.3%) demonstrated concomitant pathogenic *POLE* EDM, mismatch repair deficiency, and abnormal p53 (MMRd–*POLE*mut–p53abn ECs) (Table 1). All cancers had a known pathogenic *POLE* mutation, as described by León‐Castillo *et al* 18, including p.Pro286Arg (1), p.Val411Leu (4), p.Ser297Phe (2), p.Ser459Phe (1), p.Phe367Ser (2), p.Leu424Ile (1), and p.Met295Arg (1). These patients had a mean age of 60 years and presented with early‐stage tumours (58.3% stage IA and 33.3% stage IB). Most MMRd–*POLE*mut–p53abn ECs were endometrioid (*n* = 9, 75%), whereas two were classified as mixed ECs (serous and endometrioid) and one as serous EC. Eleven tumours were available for morphological review (supplementary material, Table S1). An abundant lymphoid infiltrate was present in these tumours [8 (72.7%) had prominent TILs and 10 (90.9%) peritumoural lymphocytes]. Hobnailing was observed in one (9.1%) tumour, slit‐like spaces were present in two (18.2%), and three (27.3%) had papillary growth. In 4/11 of these MMRd–*POLE*mut–p53abn ECs, we were able to re‐evaluate p53 and MMR IHC. All four displayed subclonal p53abn expression and two of them additionally showed subclonal loss of MLH1 with concomitant loss of PMS2 expression.

We further analysed the seven MMRd–*POLE*mut–p53abn ECs available in the TCGA. Five tumours had a *TP53* (likely) pathogenic mutation and two had a VUS. Hierarchical clustering based on SNV and SCNA proportions (supplementary material, Figure S3) showed five tumours clustering with single‐classifier *POLE*mut ECs, two with single‐classifier MMRd ECs, and none with single‐classifier p53abn ECs. Of note, the two ECs that clustered with single‐classifier MMRd cancers had a V411L and L424I *POLE* variant, respectively, with low C>A substitutions and high indels 18 and pathogenic *TP53* mutations.

The limited number of MMRd–*POLE*mut–p53abn ECs with available clinical data (*n* = 9) did not allow for survival analysis.

### Clinical outcome of multiple‐classifier ECs versus single‐classifier ECs

Finally, we investigated the outcome of MMRd–p53abn ECs and *POLE*mut–p53abn ECs in the pooled study population (supplementary material, Table S2). Fourty‐four MMRd–p53abn ECs were available for survival analysis. Patients had a 5‐year RFS and OS of 83.4% and 82%, respectively (supplementary material, Figure S4). RFS and OS could be analysed for 23 patients with *POLE*mut–p53abn ECs. The survival analysis revealed that these ECs were associated with a favourable outcome with a 90.9% 5‐year RFS and 5‐year OS of 95.2% (supplementary material, Figure S4).

**Figure 4:**
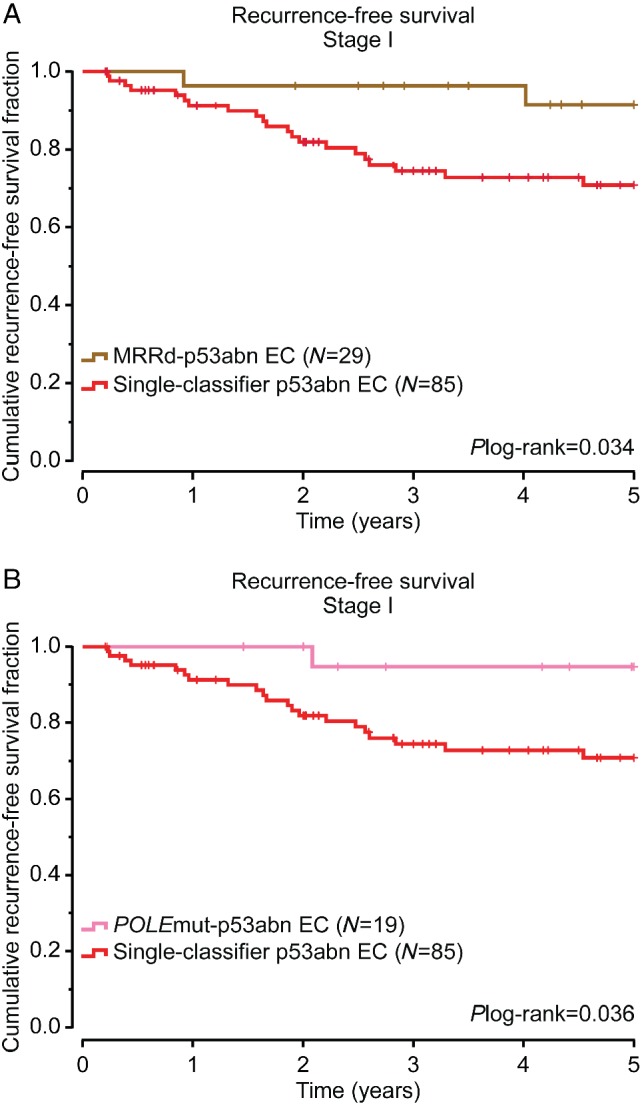
Survival analysis of multiple‐classifier EC compared with single‐classifier p53abn EC. Five‐year recurrence‐free survival analysis of stage I MMRd–p53abn EC (A) and *POLE*mut–p53abn EC (B) compared with single‐classifier p53abn EC. Two‐sided *P* values were obtained from log‐rank testing. Patients were classified based on *POLE*mut–p53abn (pink), MMRd–p53abn (brown), and single‐subtype p53abn (red).

We further analysed the clinical outcome of multiple‐classifier ECs by comparing their 5‐year RFS with single‐classifier p53abn ECs. For this purpose, we used a cohort of 187 single‐classifier p53abn ECs described previously 12, 13. These patients had a lower proportion of stage I disease (93, 49.7%) compared with MMRd–p53abn ECs (31, 70.5%) and *POLE*mut–p53abn ECs (19, 82.6%). Due to the differences in stage distribution, only stage I ECs were analysed. Thus, patients with stage I single‐classifier p53abn ECs had a 5‐year RFS of 70.8%, while those with stage I MMRd–p53abn ECs (*n* = 31) had an RFS of 92.2% (*p* = 0.024) and patients with stage I *POLE*mut–p53abn EC (*n* = 19) showed an RFS of 94.1% (*p* = 0.050) (Figure 4).

## Discussion

The molecular EC classification developed by TCGA has the potential to become the new standard in diagnostic pathology. Together with stage, the molecular subtype of EC provides powerful prognostic information; unlike stage, this information can be very accurately determined at the time of initial biopsy 33. When using the surrogate approach to determine the four molecular subtypes (*POLE*mut, MMRd, p53abn, and NSMP EC), a small but clinically relevant number of ECs (3–5%) have been unclassifiable due to the presence of more than one molecular classifying feature. These so‐called ‘multiple‐classifier’ ECs create a hurdle in the application of the molecular EC classification in practice, as it is not immediately apparent how these should be classified/treated. This issue is particularly relevant for the co‐occurrence of abnormal p53 IHC in the context of MMRd or *POLE*mut, as these features, when present singly, are associated with opposing clinical outcomes. The present study is the first to provide an extensive characterisation of these uncommon multiple‐classifier ECs and by doing so, provides guidance on how these should be interpreted.

The molecular landscape of single‐classifier *POLE*mut EC, p53abn EC or MMRd EC cases is considered to be shaped by their driver alterations, being *POLE* EDM, *TP53* mutation or MMR deficiency. Interestingly, we observed similar mutational changes and SCNA between MMRd–p53abn EC and *POLE*mut–p53abn EC with single‐classifier MMRd and *POLE*mut EC, respectively. This strongly suggests that *TP53* variants occurring in the context of an MMRd or *POLE*mut EC are likely passenger events, not affecting the molecular landscape of the tumour. This is further supported by the phenotype of the multiple‐classifier ECs, as pathology review revealed an enrichment for features associated with MMRd or *POLE*mut EC (TILs, peritumoural lymphocytes, squamous metaplasia) and not serous‐like (p53abn) features. Another important observation in support of this interpretation is the unusual high frequency (47–60%) of subclonal abnormal p53 staining in the multiple‐classifier EC. Of note, cases designated as single‐classifier p53 EC from the previously published PORTEC‐1/‐2 cohorts (*n* = 74) did not show subclonal expression 10. This same observation was made independently by Singh *et al* 15. Together, these data strongly support the interpretation that the *TP53* mutation is a later event during tumour progression in multiple‐classifier EC, without affecting the molecular landscape or the phenotype.

Multiple‐classifier EC can be identified on the basis of sequencing approaches (e.g. presence of *POLE* and *TP53* mutations by sequencing) or by the use of surrogate markers (e.g. abnormal p53 IHC). By sequencing, more *TP53* mutations will be identified, resulting in the higher number of multiple‐classifiers present in TCGA (*TP53* mutational status‐based) compared with the cases identified using the surrogate marker approach (p53 IHC‐based) 34. This is likely the result of the high number of ‘non‐hotspot’ *TP53* mutations in *POLE*mut–p53abn EC compared with single‐classifier *TP53*mut EC, as well as the higher sensitivity of sequencing approaches to identify subclonal events (low allele frequency). These non‐hotspot *TP53* mutations appear to not always impact the expression and function of p53. This is in line with Singh *et al* 15, where a high proportion of cases with such a *TP53* variant and p53 wild‐type staining pattern were identified in multiple‐classifier ECs.

We also describe a limited number of MMRd–*POLE*mut–p53abn ECs (‘triple‐classifier’). The data available are insufficient to suggest how to treat patients with these cancers. The presence of p53abn subclonal staining in these cases suggests that, as in MMRd–p53abn and *POLE*mut–p53abn ECs, the *TP53* mutations occur as a secondary event. However, it is difficult to assess, with the present data, whether *POLE*mut or MMRd are the driving events in these cancers. A larger number of these triple‐classifier cases will be required to study the biological behaviour of these ECs. Until then, considering the results reported by León‐Castillo *et al* 18 on MMRd–*POLE*mut ECs and the present study, we suggest that these triple‐classifiers be classified as *POLE*mut ECs if they have a pathogenic *POLE* EDM based on whole‐exome sequencing (WES) data or, in the case of absence of WES data, if the *POLE* EDM corresponds to one of the 11 pathogenic mutations described by León‐Castillo *et al* 18. In tumours in which the triple‐classifier ECs carry a *POLE* EDM that does not comply with the previous criteria, we recommend that they be classified as MMRd ECs, as discussed in depth by León‐Castillo *et al* 18.

Finally, we addressed the question of whether the biological behaviour of *POLE*mut and MMRd ECs is impacted by the presence of abnormal (subclonal) p53 expression. Although the number of cases is limited, the clinical outcomes of MMRd–p53abn ECs and *POLE*mut–p53abn ECs are strikingly different from what would be expected in single‐classifier p53abn (SCNA‐high/serous‐like) ECs 1, 9, 10, 11, 12, 13, 14. This supports the concept that passenger events do not affect biological behaviour. For clinical management, this means that the presence of *TP53* mutations in the context of MMRd EC or *POLE*mut EC should not prompt intensified treatment.

In addition to multiple‐classifier ECs with mutant p53 expression/*TP53* mutation, a fourth group of ECs with multiple classifying features can be encountered: MMRd–*POLE*mut ECs. The genomic architecture and clinical outcome of MMRd–*POLE*mut ECs differ depending on the pathogenicity of the *POLE* exonuclease domain variant and the ultramutated phenotype it confers to the tumour 18. Thus, the base change and indel proportion of ECs with pathogenic *POLE* EDM (as defined by the POLE score) and MSI are similar to MSS ECs with pathogenic *POLE* EDM 18. Additionally, when examining a cohort of MMRd ECs with pathogenic *POLE* EDM, although the number of patients was limited (*n* = 14), a good clinical outcome was observed (5‐year RFS 92.3%), in line with the prognosis described previously for single‐classifier *POLE*mut ECs (supplementary material, Figure S5) 9, 10, 11, 12, 13, 14, 18, 35, 36. These findings support the classification of tumours with a pathogenic *POLE* EDM and MMRd as single‐classifier *POLE*mut ECs.

In conclusion, this study is the first to describe evidence in support of categorising multiple‐classifier *POLE*mut–p53abn EC as single‐classifier *POLE*mut, and MMRd–p53abn EC as single‐classifier MMRd. Although rare (∼3%), correct designation of multiple‐classifier ECs facilitates the implementation of the molecular EC classification, enabling them to be included in future studies and, more importantly, providing valuable information for clinicians and patients to guide management.

## Author contributions statement

AL and EG carried out experiments and analysed data. TB, CBG, DNC, JNM, and RN conceived experiments and analysed data. VTHBMS, MM, SK, SYB, JWC, EE, TTR, RAS, RG, XM, EO, and BTH analysed data. All the authors were involved in writing the paper and had final approval of the submitted and published versions.

## Supporting information


**Supplementary figure legends**

**Figure S1.** Heatmap showing hierarchical clustering of MMRd–p53abn, single‐classifier MMRd, and single‐classifier p53abn ECs in TCGA

**Figure S2.** Heatmap showing hierarchical clustering of *POLE*mut–p53abn, single‐classifier *POLE*mut, and single‐classifier p53abn ECs in TCGA

**Figure S3.** Heatmap showing hierarchical clustering of MMRd–*POLE*mut–p53abn, single‐classifier MMRd, single‐classifier *POLE*mut, and single‐classifier p53abn ECs in TCGA

**Figure S4.** Overall survival and recurrence‐free survival of MMRd–p53abn and *POLE*mut–p53abn ECs

**Figure S5.** Clinical outcome of MMRd–*POLE*mut ECs

**Table S1.** Morphological features of MMRd–p53abn, *POLE*mut–p53abn, and MMRd–*POLE*mut–p53abn ECs

**Table S2.** Clinicopathological features of MMRd–p53abn and *POLE*mut–p53abn ECs with available survival data
