# Supplementary material for: Clinicopathological and molecular characterisation of ‘multiple‐classifier’ endometrial carcinomas
Source: J Pathol. 2020 Jan 12;250(3):312–22. doi: 10.1002/path.5373 (PMC7065184; doi:10.1002/path.5373)
Supplement: Supplementary file 1 — Supplementary figure legends [file PATH-250-312-s008.docx]

**Clinicopathological and molecular characterisation of ‘multiple-classifier’ endometrial carcinomas**

León-Castillo *et al. J Pathol* DOI: 10.1002/path.5373

**Supplementary figure legends**

**Figure S1.** Heatmap showing hierarchical clustering of MMRd–p53abn, single-classifier MMRd, and single-classifier p53abn ECs in TCGA. Based on copy number changes (A) and mutational changes (B) analysed separately. Patients were classified into groups based on MMRd–p53abn (brown), single-classifier MMRd (green), and single-classifier p53abn (red). Samples were ordered based on hierarchical clustering.

**Figure S2.** Heatmap showing hierarchical clustering of *POLE*mut–p53abn, single-classifier *POLE*mut, and single-classifier p53abn ECs in TCGA. Based on copy number changes (A) and mutational changes (B) analysed separately. Patients were classified into groups based on *POLE*mut–p53abn (pink), single-classifier *POLE*mut (blue), and single-classifier p53abn (red). Samples were ordered based on hierarchical clustering.

**Figure S3.** Heatmap showing hierarchical clustering of MMRd–*POLE*mut–p53abn, single-classifier MMRd, single-classifier *POLE*mut, and single-classifier p53abn ECs in TCGA. Based on mutational changes and copy number changes. Individual similarity metrics for copy number and mutational changes were combined using Euclidean distance. Patients were classified into groups based on MMRd–*POLE*mut–p53abn ECs (black), single-classifier MMRd ECs (green), single-classifier *POLE*mut ECs (blue), and single-classifier p53abn ECs (red). Samples were ordered based on hierarchical clustering.

**Figure S4.** Overall survival and recurrence-free survival of MMRd–p53abn (A and B, respectively) and *POLE*mut–p53abn ECs (C and D).

**Figure S5.** Clinical outcome of MMRd–*POLE*mut ECs. Kaplan–Meier survival curves of RFS (A) and OS (B) of MMRd–*POLE*mut ECs with a pathogenic *POLE* EDM.
