# Supplementary figures and images for: Clinicopathological and molecular characterisation of ‘multiple‐classifier’ endometrial carcinomas
Source: J Pathol. 2020 Jan 12;250(3):312–22. doi: 10.1002/path.5373 (PMC7065184; doi:10.1002/path.5373)

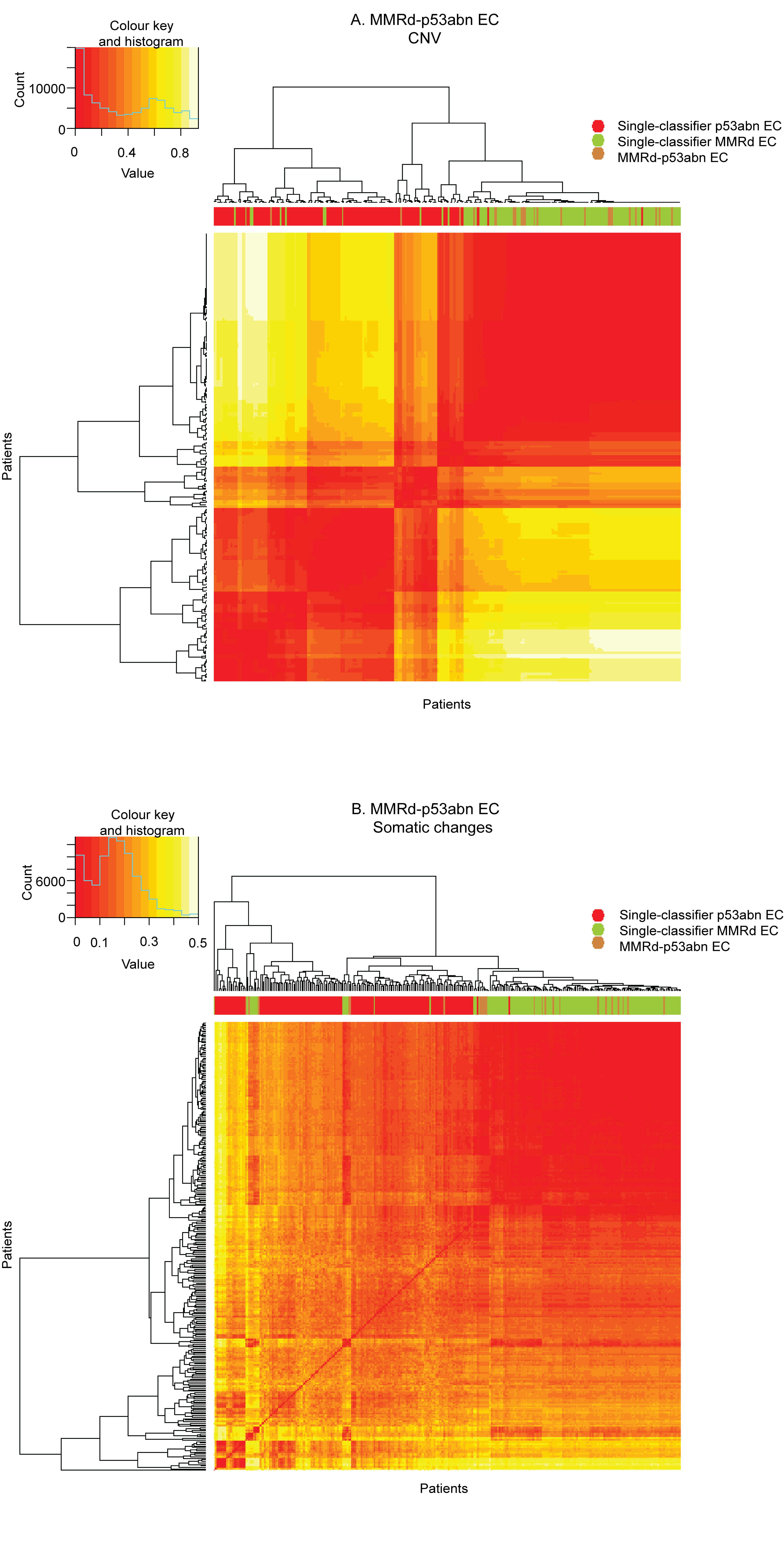

Supplement: Supplementary file 2 — Figure S1. Heatmap showing hierarchical clustering of MMRd–p53abn, single‐classifier MMRd, and single‐classifier p53abn ECs in TCGA [file PATH-250-312-s001.tif]

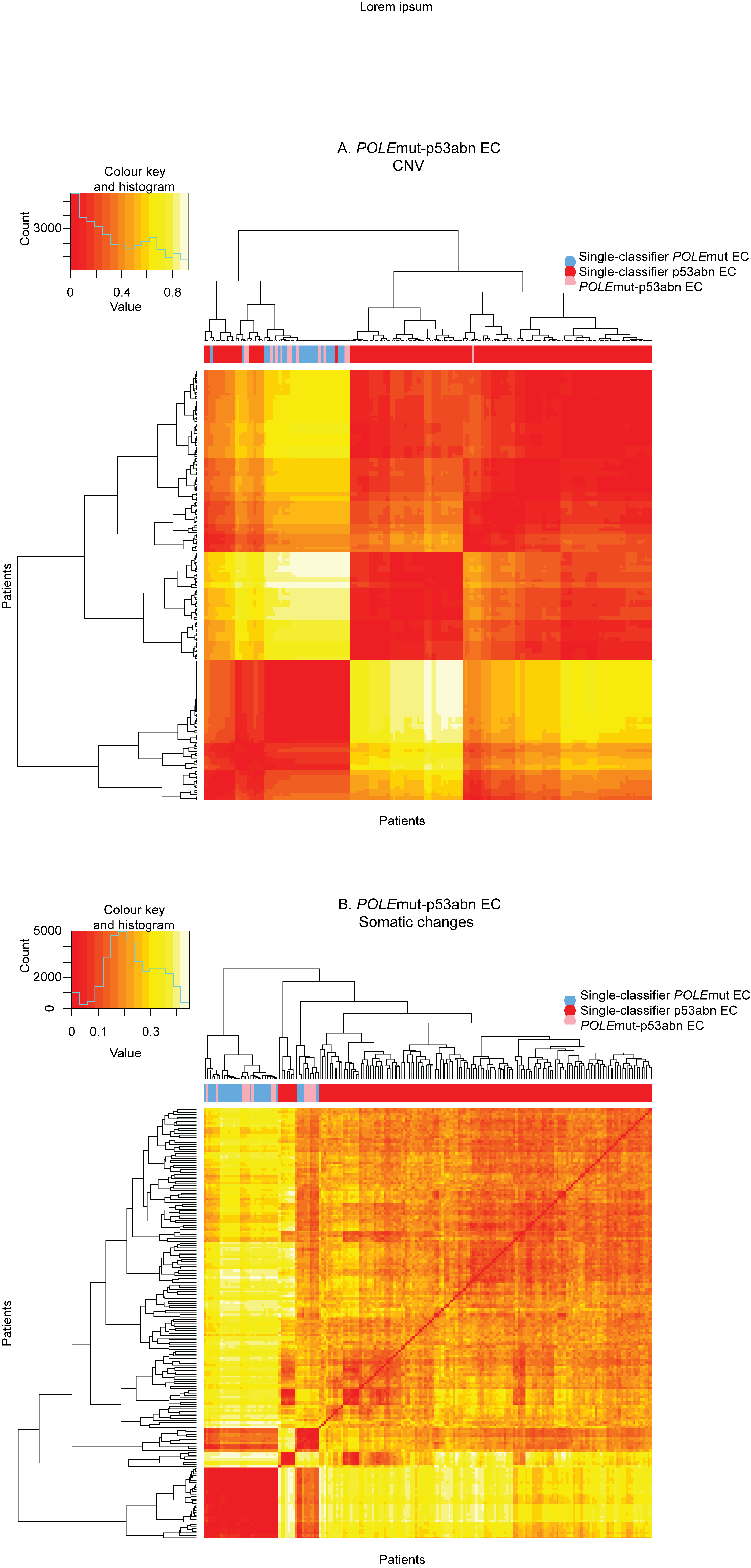

Supplement: Supplementary file 3 — Figure S2. Heatmap showing hierarchical clustering of POLEmut–p53abn, single‐classifier POLEmut, and single‐classifier p53abn ECs in TCGA [file PATH-250-312-s002.tif]

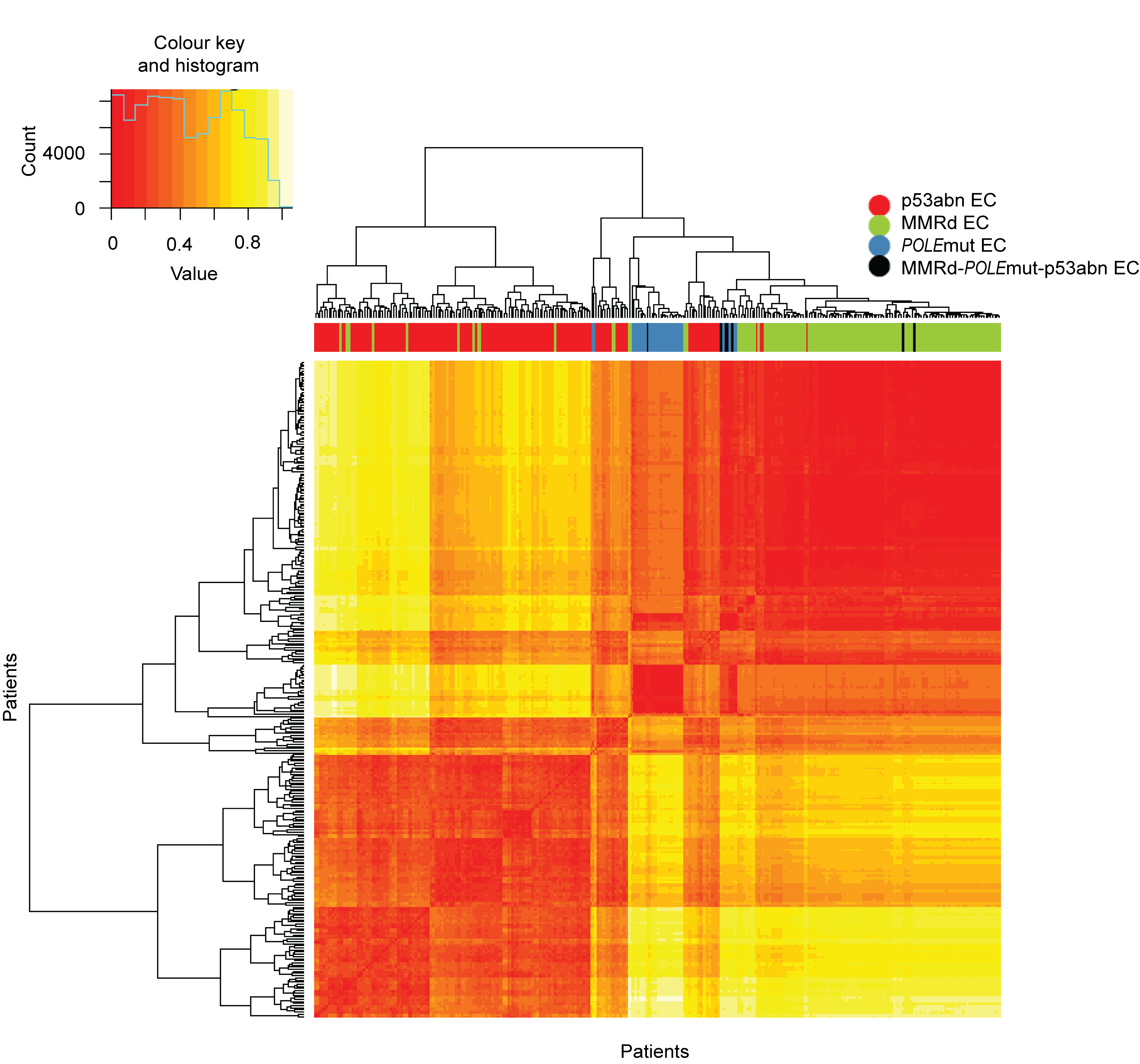

Supplement: Supplementary file 4 — Figure S3. Heatmap showing hierarchical clustering of MMRd–POLEmut–p53abn, single‐classifier MMRd, single‐classifier POLEmut, and single‐classifier p53abn ECs in TCGA [file PATH-250-312-s003.tif]

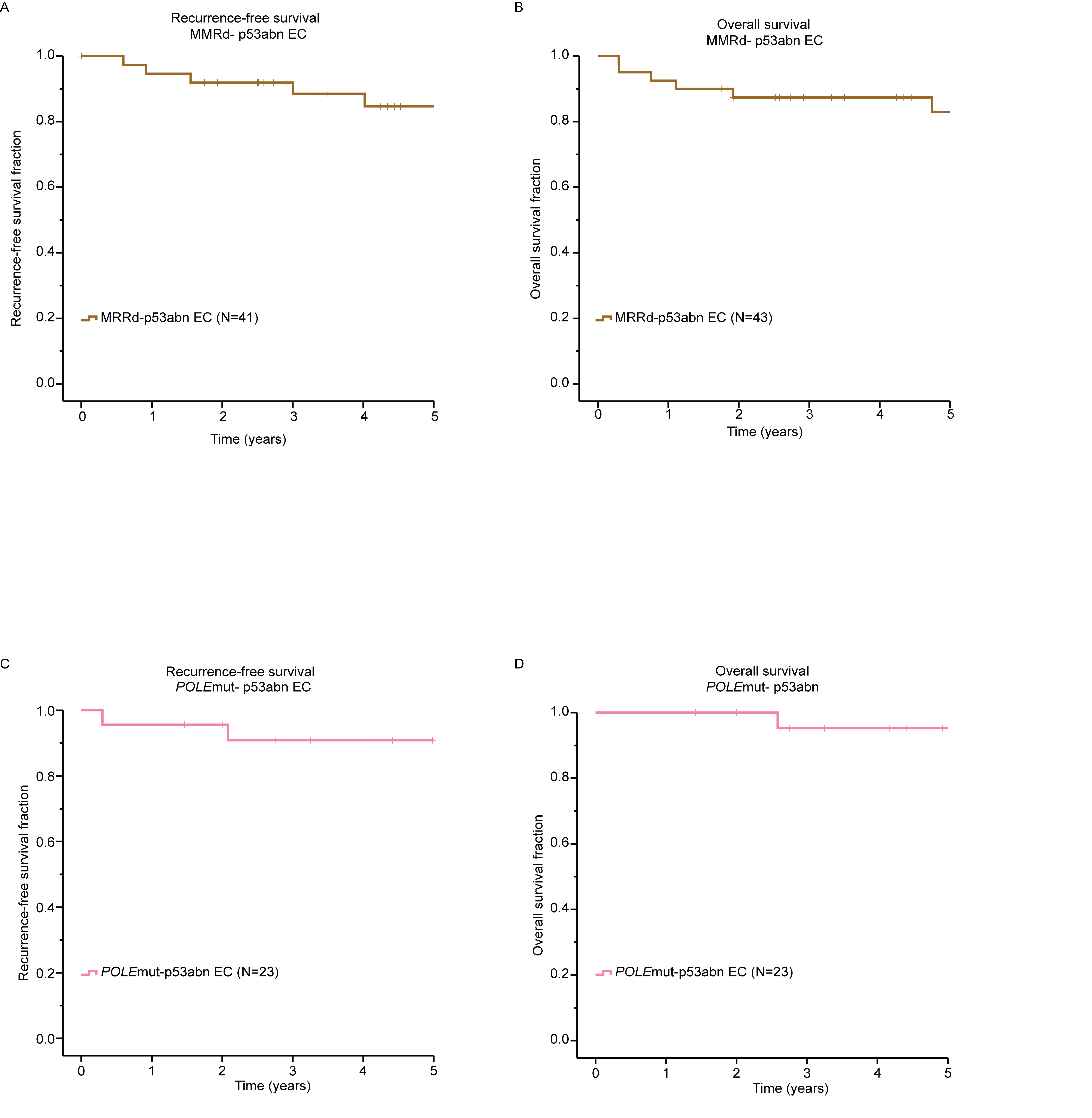

Supplement: Supplementary file 5 — Figure S4. Overall survival and recurrence‐free survival of MMRd–p53abn and POLEmut–p53abn ECs [file PATH-250-312-s004.tif]

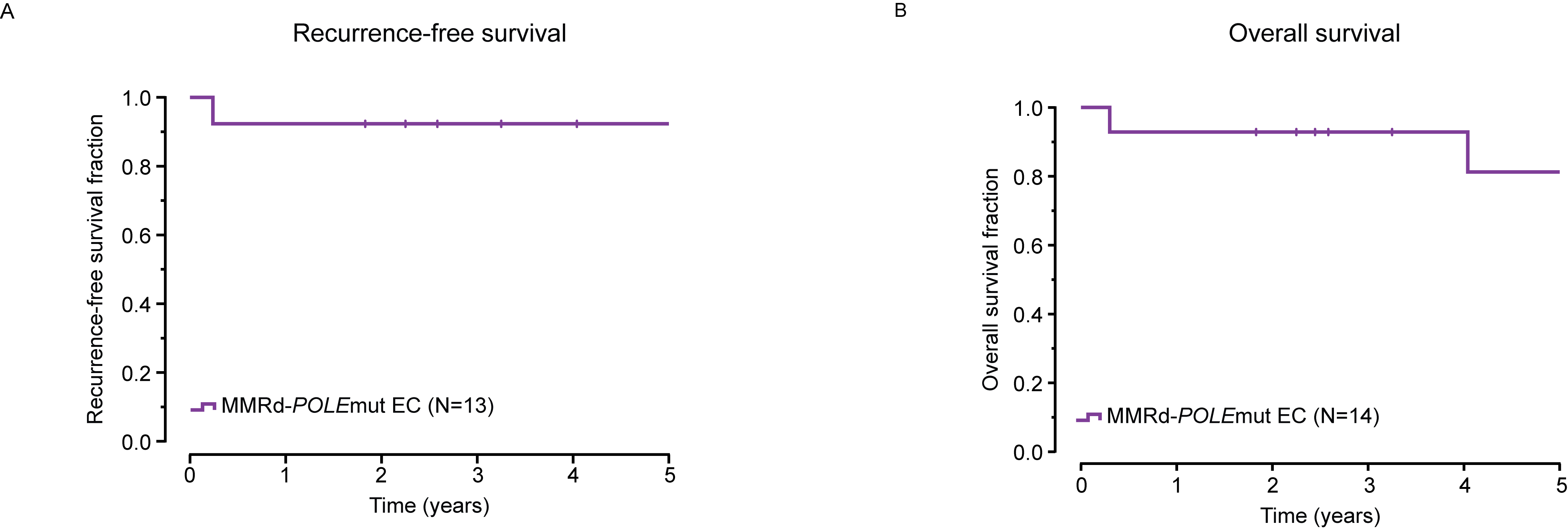

Supplement: Supplementary file 6 — Figure S5. Clinical outcome of MMRd–POLEmut ECs [file PATH-250-312-s005.tif]
